# Pulmonary responses following cardiac rehabilitation and the relationship with functional outcomes in children and young adults with heart disease

**DOI:** 10.3389/fsurg.2024.1356501

**Published:** 2024-05-20

**Authors:** Cassidy E. Kershner, William D. Hardie, Clifford Chin, Alexander R. Opotowsky, Elizabeth B. Aronoff, Wayne A. Mays, Sandra K. Knecht, Adam W. Powell

**Affiliations:** ^1^ Department of Pediatrics, University of Cincinnati College of Medicine, Cincinnati, OH, United States; ^2^ Department of Pediatric Pulmonology, Cincinnati Children’s Hospital Medical Center, Cincinnati, OH, United States; ^3^ The Heart Institute, Cincinnati Children’s Hospital Medical Center, Cincinnati, OH, United States

**Keywords:** cardiac rehabilitation, cardiopulmonary exercise testing, CHD (congenital heart disease), pulmonary function, exercise therapy

## Abstract

**Introduction:**

Patients with congenital heart disease (CHD) often have pulmonary abnormalities and exercise intolerance following cardiac surgery. Cardiac rehabilitation (CR) improves exercise capacity in patients with CHD, but minimal study has been performed to see if resting and dynamic pulmonary performance improves following CR in those with prior cardiac surgery.

**Methods:**

This was a retrospective cohort study of all patients who completed ≥12 weeks of CR from 2018 through 2022. Demographic, cardiopulmonary exercise test (CPET), spirometry, 6-minute walk, functional strength measures, and outcomes data were collected. Data are presented as median[IQR]. A Student's *t*-test was used for comparisons between groups and serial measurements were measured with a paired *t*-test. A *p* < 0.05 was considered significant.

**Results:**

There were a total of 37 patients [age 16.7 (14.2–20.1) years; 46% male] included. Patients with prior surgery (*n* = 26) were more likely to have abnormal spirometry data than those without heart disease (*n* = 11) (forced vital capacity [FVC] 76.7 [69.1–84.3]% vs. 96.4 [88.1–104.7]%, *p* = 0.002), but neither group experienced a significant change in spirometry. On CPET, peak oxygen consumption increased but there was no change in other pulmonary measures during exercise. Percent predicted FVC correlated with hand grip strength (*r* = 0.57, *p* = 0.0003) and percent predicted oxygen consumption (*r* = 0.43, *p* = 0.009). The number of prior sternotomies showed negative associations with both percent predicted FVC (*r* = −0.43, *p* = 0.04) and FEV_1_ (*r* = −0.47, *p* = 0.02).

**Discussion:**

Youth and young adults with a prior history of cardiac surgery have resting and dynamic pulmonary abnormalities that do not improve following CR. Multiple sternotomies are associated with worse pulmonary function.

## Introduction

Cardiac surgery is associated with reduced fitness and impaired pulmonary function (1, 2). Cardiac rehabilitation (CR) is widely used in the rehabilitation of adults with acquired cardiac disease but has minimally been used in pediatric facilities for children and young adults with congenital heart disease (CHD). Cardiopulmonary fitness is a major factor for future disability and all-cause mortality and is the target of improvement for CR programs for those with CHD (3). Despite the lack of widespread usage, CR has been shown to improve fitness in multiple heart disease populations including patients with CHD (4, 5).

While CR has been shown to improve aerobic fitness and musculoskeletal strength, there has been minimal research exploring the impact of CR on resting spirometry and on ventilatory function during exercise (6). Previous studies demonstrate exercise training leads to improvements in lung volumes and flows in healthy children and adults as well as individuals with chronic lung diseases such as emphysema and asthma (7–9). Inhalation training has also been shown to increase dynamic lung volumes and slow deconditioning in peak VO­_2_ in patients with tetralogy of Fallot or a Fontan circulation (10, 11). It is unclear if CR delivered in a clinical as opposed to a research environment results in similar changes. Additionally, there has been minimal research into how baseline pulmonary function relates to functional CR outcomes, including handgrip, 6-minute walk distance, and sit-to-stand repetitions. Lastly, while median sternotomy is a known risk factor for future restrictive patterns of spirometry, there has been minimal study into the relationship between multiple median sternotomies and CR outcomes.

The aims of this study were: (1) to evaluate the effect of CR on the resting pulmonary function and dynamic components of ventilation in children and young adults enrolled in a clinical CR program; (2) to determine the relationship between resting and dynamic pulmonary function and functional outcomes measured during CR; and (3) to describe the relationship between multiple median sternotomies and pulmonary function, exercise performance, and CR outcomes.

## Materials and methods

This was a retrospective cohort study of pediatric and adult patients with cardiac disease who have completed at least 12 weeks of CR at Cincinnati Children's Hospital Medical Center (CCHMC) from January 2018 to December 2022. Data on enrollment date, number of completed weeks and sessions, and information on program completion were collected. All patients underwent cardiopulmonary exercise testing (CPET) with baseline resting spirometry within 45 days of beginning and finishing CR. Additional data collected included patients’ demographic information, medical diagnosis, and prior surgical history. Exclusion criteria included previous enrollment in the CR program, failure to complete at least 12 weeks of the CR program, submaximal effort CPET, and testing using a treadmill. Patients were further excluded from pulmonary function subanalysis for uninterpretable spirometry.

CR was performed per the standard of the American Association of Cardiovascular and Pulmonary Rehabilitation and has been previously described (12). Participants attended two to three 1-hour sessions each week for a total of at least 12 weeks. Training occurred either at the CCHMC cardiac rehabilitation gym, through virtual encounters, or combined in-person and virtual sessions. CR outcomes recorded at baseline and program completion included physical body measurements and performance measures.

All cardiopulmonary exercise tests (CPETs) were conducted on a stationary cycle ergometer (Corival; Lode; Groningen, The Netherlands) with an individualized incremental ramp protocol. Individualized protocols were chosen by the exercise physiologist based on the patient body surface area and expected fitness levels. Each session was targeted to last approximately 10 min. Cardiopulmonary responses to exercise were assessed breath-by-breath (Ultima CardiO2; MGC Diagnostics; Saint Paul, MN, USA). Maximal effort criteria were designated for tests with two of the three criteria met: respiratory exchange ratio >1.10, predicted peak heart rate ≥85%, or subjective exhaustion. Predicted peak oxygen consumption (VO_2_) was calculated per Wasserman et al. and Cooper et al. (13, 14). The ventilatory anaerobic threshold (VAT) was determined non-invasively using the modified V-slope method (15).

All patients underwent resting spirometry before exercise to aid in the CPET interpretation. Spirometry was assessed using a metabolic cart (TrueMax 2400; Parvo Medics; Salt Lake City, UT, USA or Ultima CardiO2; Medgraphics; St. Paul, MN, USA). Spirometry was performed per American Thoracic Society recommendations and has been previously described (16). A pediatric pulmonologist with over 25 years of experience (WDH) overread spirometry to meet acceptable and repeatable criteria and interpreted each test blinded to clinical history. Spirometry was interpreted as normal if endpoints fell within the 95% confidence intervals (95% CI) of published normative standards (17, 18). Tests were interpreted as suggestive of restriction if the forced vital capacity (FVC) and forced expiratory volume at one second (FEV_1_) were reduced and the FEV_1_/FVC normal. Spirometry was interpreted as obstructive if meeting all four of the following criteria: the FEV_1_/FVC was reduced, the forced expiratory flow at 25%−75% of the FVC (FEF_25–75_) was reduced, evidence of coving (defined as concavity on expiratory limb of the flow-volume curve), and failure to plateau (defined as volume change of <25 ml/s at the end of exhalation). Maximal voluntary ventilation (MVV) was calculated as FEV_1 _× 40 (19). Breathing reserve was calculated as MVV—maximal exercise ventilation (13).

## Statistical analyses

Descriptive statistics were denoted as median [interquartile range]. Primary analysis utilized two-tailed paired *t*-tests to compare baseline and final cardiopulmonary outcomes. For sub-analysis, Spearman correlations were conducted between both the percent of predicted FVC and FEV_1_ and functional outcomes. All *t*-tests and correlations were considered significant with a *p*-value <0.05. Statistical analyses were performed using JMP®, Version 16 from SAS Institute Inc. (Cary, NC).

## Results

**Figure 1:**
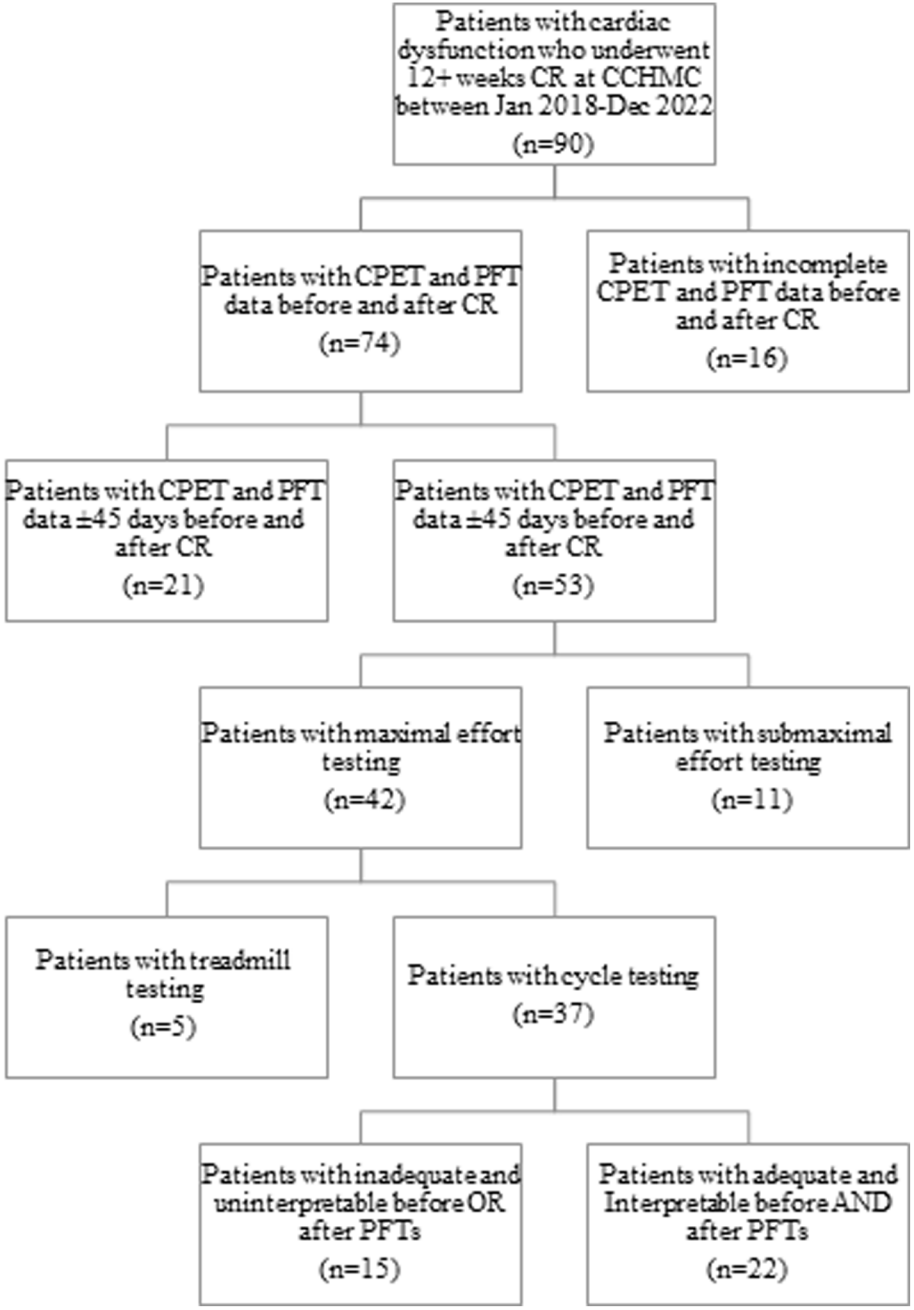
Flowsheet of all patients enrolled in cardiac rehabilitation demonstrating how the use of our inclusion/exclusion criteria resulted in our final cohort. CCHMC, Cincinnati Children's Hospital Medical Center; CPET, cardiopulmonary exercise test; spirometry, pulmonary function testing; CR, cardiac rehabilitation.

A total of 90 patients were enrolled in CR at Cincinnati Children's Hospital Medical Center from January 2018 to December 2022. Out of the total patients enrolled, 37 patients (41.1%) finished the full course of CR and had a pair of maximal CPETs. Of those 37 patients, 29 patients (59.4%) had interpretable spirometry results). Patients were excluded from analysis if they did not undergo both spirometry and CPET within 45 days of starting and finishing CR (*n* = 37) and for testing using a treadmill (*n* = 5). Patients with submaximal effort testing had their baseline PFT data but not their CPET data included for analysis (*n* = 11). Patients with uninterpretable pulmonary function testing did not have their PFT data analyzed but did have the CPET and CR outcome data included (*n* = 15) (Figure 1). The demographics of the cohort are described in Table 1.

**Table 1:** Baseline demographics of those who completed cardiac rehabilitation.

Age (years)	16.7 [14.2–20.1]
<21	29 (78.4%)
≥21	8 (21.6%)
Sex	
Female	20 (54.1%)
Male	17 (45.9%)
Race	
White	27 (73.0%)
African-American	7 (18.9%)
Asian	1 (2.7%)
Other	2 (5.4%)
Program type	
In Person	26 (70.3%)
Virtual	6 (16.2%)
Hybrid	5 (13.5%)
Diagnosis	
Fontan	8 (21.6%)
HT	6 (24.3%)
CM	5 (13.5%)
ToF	4 (10.8%)
Syncope	3 (8.1%)
Truncus arteriosus	2 (5.4%)
D-TGA	1 (2.7%)
Heart block	1 (2.7%)
CDH	1 (2.7%)
Exercise intolerance	1 (2.7%)
Interrupted AA	1 (2.7%)
Shone complex	1 (2.7%)
Subaortic membrane	1 (2.7%)
Scimitar syndrome	1 (2.7%)
Status post Kawashima	1 (2.7%)
Total previous sternotomies	2.5 [2.0–4.0]
Time since last sternotomy (months)	10.5 [0.2–14.0]

Data are presented as median[IQR] or number of patients (percentage of total). For variables with missing data, the number of patients with available data is provided. Single ventricle physiology s/p a Fontan palliation (Fontan); HT, heart transplant; CM, cardiomyopathy; ToF, tetralogy of Fallot; D-TGA, dextro-transposition of the great arteries; CDH, congenital diaphragmatic hernia; AA, aortic arch (AA).

Results of pre and post-testing for CR can be seen in Table 2. The resting spirometry was abnormal in most patients with 12 patients having a pattern suggestive of restriction, 3 with an obstructive pattern, and 1 mixed. The remainder had normal spirometry (13/29; 48%). These were similar on the spirometry following CR: restrictive (*n* = 11), obstructive (*n* = 3), mixed (*n* = 2), and normal (*n* = 13). There were 4 patients who had either their resting or final spirometry deemed uninterpretable with the interpretable results included in the analysis, resulting in the spirometry number discrepancy.

**Table 2:** Body composition, spirometry, cardiopulmonary exercise test, and functional outcomes for the patients who completed cardiac rehabilitation.

Demographics (*n* = 37)	Pre	Post	*p* value
Weight (kg)	62.3 [48.8–71.4]	66.2 [48.6–75.4]	0.06
Height (cm)	164.3 [153.4–172.6]	164.0 [154.5–175]	0.01
BMI (kg/m^2^)	22.7 [19.7–26.8]	22.8 [19.3–27.2]	0.40
PFT variables			
FVC (%) (*n* = 28)	84.0 [69.0–99.50]	86.0 [73.5–99.0]	0.2
FEV_1_ (%) (*n* = 25)	86.0 [66.0–99.5]	81.0 [73.5–96.5]	0.9
FEV_1_/FVC (%) (*n* = 22)	84.8 [80.9–91.0]	80.0 [74.5–96.5]	0.2
FEF_25−75_ (%) (*n* = 28)	76.0 [60.5–94.0]	81.0 [58.5–97.0]	0.9
Peak breathing reserve (*n* = 25)	34.0 [15.5–60.0]	36.0 [22.0–50.5]	0.8
CPET variables (*n* = 37)			
Peak RER	1.3 [1.2–1.3]	1.3 [1.2–1.3]	0.6
Peak workload (watts)	107.0 [87.5–144.0]	118.0 [93.5–162.5]	<0.0001
VAT (%)	45.0 [40.0–50.5]	53.0 [39.0–57.0]	0.07
Peak VO_2_ (mL/min)	1,586.0 [1,216.5–1,795.5]	1,651.0 [1,252.0–1,994.0]	0.03
Peak VO_2_ (% predicted)	68.0 [55.0–74.0]	71.0 [59.5–81.5]	0.02
VE/VCO_2_ slope (peak)	34.7 [31.0–38.5]	36.6 [31.0–39.2]	0.8
VE/VCO_2_ slope (VAT)	30.0 [26.7–33.5]	28.0 [24.8–33.7]	0.2
Baseline respiratory rate (bpm)	23.5 [20.0–27.8]	27.0 [23.5–30.5]	0.002
Peak respiratory rate (bpm)	50.0 [42.3–61.8]	52.0 [40.0–59.0]	0.8
Rest tidal volume (L)	0.74 [0.52–0.92]	0.66 [0.52–0.83]	0.1
Peak tidal volume (L)	1.29 [0.89–1.74]	1.36 [1.03–1.79]	0.4
Rest minute ventilation (L/min)	15.8 [12.7–21.3]	17.1 [14.6–20.8]	0.3
Peak minute ventilation (L/min)	67.5 [50.3–80.5]	64.5 [54.2–80.1]	0.1
6 Min walk (*n* = 36)			
Distance walked (m)	465.0 [405.0–507.5]	530.0 [490.0–600.0]	<0.0001
Strength and flexibility (*n* = 36)			
Arm curl (reps)	20.0 [18.0–21.8]	23.0 [21.0–28.0]	<0.0001
Sit to stand (reps)	18.5 [14.3–21.8]	24.0 [18.0–31.0]	<0.0001
Sit & reach (cm)	38.1 [28.4–43.2]	41.4 [33.0–50.8]	<0.0001
Hand grip (*n* = 36)			
Peak right (kg)	11.1 [9.1–17.2]	12.2 [9.5–16.3]	0.02
Peak left (kg)	10.4 [8.4–15.3]	11.8 [9.1–16.3]	0.03
Peak dominant hand (kg)	11.1 [9.1–17.2]	12.2 [9.5–16.3]	0.03

Data are presented as median [IQR]. A paired *t*-test was performed to evaluate for change between tests. *P*-value <0.05 was considered significant. KG, Kilograms; CM, centimeters; BMI, body mass index; FVC, forced vital capacity; FEV_1_, forced expiratory volume at one second; FEF_25−75_, forced expiratory flow at 25%–75% of the FVC; MVV, maximal voluntary ventilation; M, meters; VO_2_, oxygen consumption; ML, milliliters; min, minute; RER, respiratory exchange ratio; VAT, ventilatory anaerobic threshold; VE/VCO_2_ slope peak, minute ventilation/carbon dioxide production slope calculated from the start to the end of exercise; VE/VCO_2_ slope VAT, minute ventilation/carbon dioxide production slope calculated from the start to the anaerobic threshold; bpm, breaths per minute; L, liters; m, meters; reps, repetitions.

The only difference in pulmonary endpoints on CPET before and after CR was the resting respiratory rate (23.5[20.0–27.8] bpm vs. 27.0 [23.5–30.5] bpm; *p* = 0.002) (Table 2). There were no significant changes in peak respiratory rate, resting or peak ventilatory equivalents, resting or peak tidal volume, resting or peak minute ventilation, or the minute ventilation/carbon dioxide production slope (VE/VCO_2_ slope).

On CPET for the entire cohort, there was a significant increase in the percent of predicted peak VO_2_ (68.0 [55.0–74.0]% vs. 71.0 [59.5–81.5]%; *p* = 0.02). On functional outcome testing, there were significant improvements in dominant hand grip strength (11.1 [9.1–17.2] kg vs. 12.2 [9.5–16.3] kg; *p* = 0.02), 6-minute walk distance (465.0 [405.0–507.5] m vs. 530.0 [490.0–600.0] m; *p* < 0.0001), sit-to-stand (18.5 [14.3–21.8] reps vs. 24.0 [18.0–31.0] reps; *p* < 0.0001), arm curls (20.0 [18.0–21.8] reps vs. 23.0 [21.0–28.0] reps; *p* < 0.0001), and sit and reach (38.1 [28.4–43.2] cm vs. 41.4 [33.0–50.8] cm; *p* < 0.0001).

**Figure 2:**
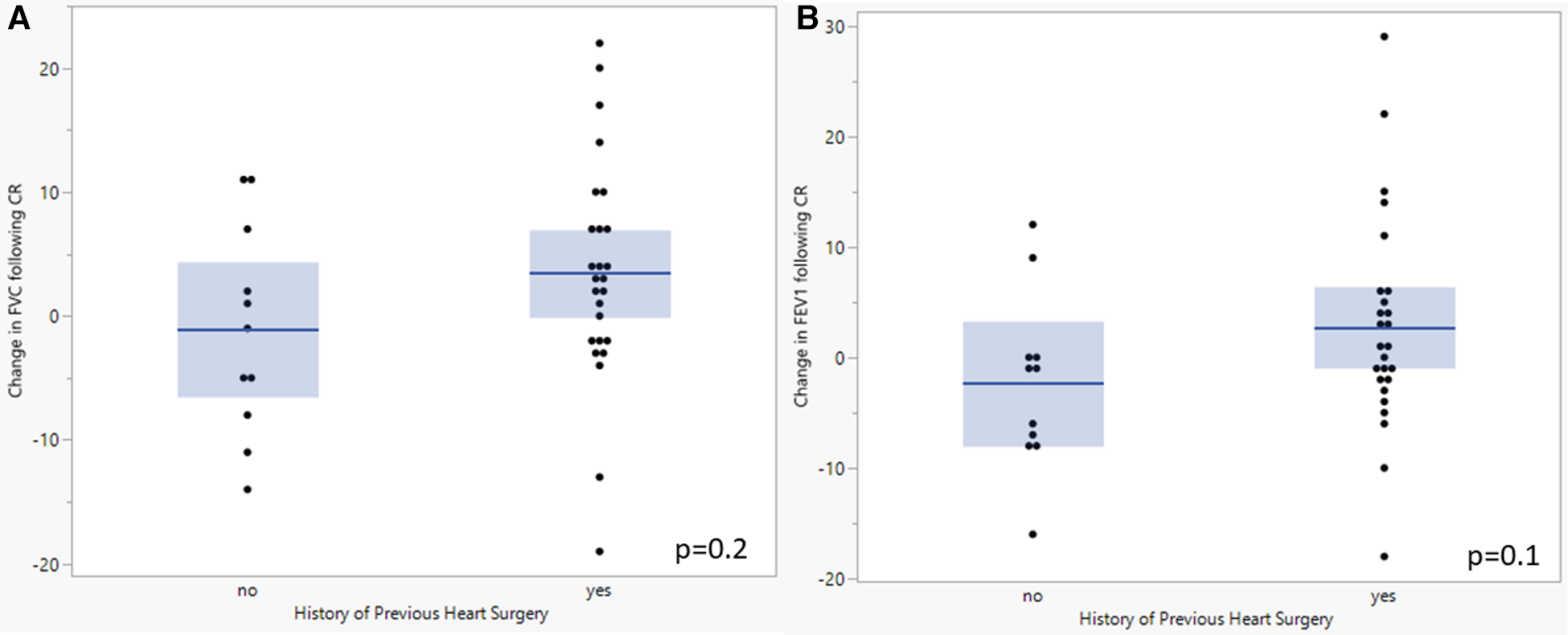
Comparison of baseline and final FVC (**A**) and FEV_1_ (**B**) following completion of cardiac rehabilitation in patients with and without previous cardiac surgery. A student's *t*-test was performed to evaluate for change between tests. *P*-value <0.05 was considered significant. FVC, Forced vital capacity; FEV_1_, forced expiratory volume at one second; CR, cardiac rehabilitation.

When comparing the baseline testing in those CR patients with previous cardiac surgery with a median sternotomy (*n* = 26) and those who have never had surgery (*n* = 11), there were significant differences in FVC (76.7 [69.1–84.3]% vs. 96.4 [88.1–104.7]%; *p* = 0.002), FEV_1_ (74.8 [68.3–81.3]% vs. 94.0 [85.0–103.0]%; *p* = 0.001), and peak breathing reserve (28.0 [17.1–38.9] L vs. 62.9 [47.9–77.9] L; *p* = 0.0006) (Table 3). With functional outcomes, there was a significant difference in 6-minute walk distance (439.6 [408.2–471.0] m vs. 513.9 [465.1–562.7] m; *p* = 0.01) but not peak VO_2_ or sit-to-stand. There were no changes in resting lung volumes in those with and without prior cardiac surgery (Figure 2) nor was there a change other CPET or functional outcomes following CR. There were no clinically significant changes in baseline PFT results from the start to completion of CR.

**Table 3:** Baseline spirometry, cardiopulmonary exercise test, and functional outcomes for patients with and without previous cardiac surgery who completed cardiac rehabilitation.

	Prior CV surgery	No prior CV surgery	*p* value
PFT variables	*N* = 19	*N* = 10	
FVC (%)	73.0 [63.0–86.0]	98.0 [86.5–103.5]	0.0006
FEV_1_ (%)	73.0 [64.0–86.0]	99.0 [83.5–103.5]	0.0004
FEV_1_/FVC (%)	91.6 [81.0–91.6]	84.3 [79.6–88.1]	0.5
FEF_25−75_ (%)	74.0 [59.0–88.0]	89.0 [64.0–97.5]	0.06
MVV	86.4 [76.0–113.6]	130.8 [91.2–163.6]	0.003
Peak breathing reserve	28.0 [14.0–36.5]	67.0 [37.3–91.0]	0.0008
CPET variables	*N* = 26	*N* = 11	
Peak RER	1.2 [1.2–1.3]	1.3 [1.2–1.3]	0.3
Peak workload (watts)	101.0 [82.0–138.5]	133.0 [106.0–167.0]	0.02
VAT (%)	46.0 [42.2–53.3]	47.0 [40.0–49.0]	0.9
Peak VO_2_ (ml/min)	1,449 [1,086–1,694.5]	1,750 [1,433–2,128]	0.1
Peak VO_2_ (% predicted)	67.0 [53.8–74.0]	71.0 [67.0–78.0]	0.08
VE/VCO_2_ slope (peak)	35.7 [32.3–39.8]	32.4 [30.7–34.7]	0.07
VE/VCO_2_ slope (VAT)	30.4 [27.8–33.3]	28.0 [22.0–33.0]	0.3
Baseline respiratory rate (bpm)	24.0 [21.9–29.0]	19.0 [16.0–24.0]	0.003
Peak respiratory rate (bpm)	55.0 [45.5–64.5]	45.0 [40.0–57.0]	0.2
Rest tidal volume (L)	0.69 [0.49–0.92]	0.74 [0.63–0.94]	0.3
Peak tidal volume (L)	1.2 [0.8–1.7]	1.5 [1.0–1.8]	0.2
Rest VE (L/min)	15.8 [12.8–22.3]	15.9 [12.6–17.9]	0.5
Peak VE (L/min)	59.2 [49.7–75.0]	71.5 [60.4–83.4]	0.4
6 Minute walk	*N* = 26	*N* = 11	
Distance walked (m)	450.0 [386.3–491.3]	505.0 [478.6–562.5]	0.01
Strength and flexibility	*N* = 26	*N* = 11	
Arm curl (reps)	18.5 [17.8–21.0]	20.5 [19.0–23.8]	0.3
Sit to stand (reps)	18.0 [13.8–22.8]	19.0 [10.4–20.5]	0.9
Sit & reach (cm)	15.0 [12.0–17.0]	12.5 [11.4–20.0]	0.8
Hand grip	*N* = 26	*N* = 11	
Peak dominant hand (kg)	24.5 [17.5–38.0]	26.5 [21.5–48.5]	0.1

Data are presented as median [IQR]. A student's *t*-test was performed to evaluate for change between tests. *P*-value <0.05 was considered significant. PFT, Pulmonary function testing; FVC, forced vital capacity; FEV_1_, forced expiratory volume at one second; FEF_25−75_, forced expiratory flow at 25%-75% of the FVC; MVV, maximal voluntary ventilation; CPET, cardiopulmonary exercise test; RER, respiratory exchange ratio; VAT, ventilatory anaerobic threshold; VO_2_, oxygen consumption; ML, milliliters; min, minute; VE/VCO_2_ slope peak, minute ventilation/carbon dioxide production slope calculated from the start to the end of exercise; VE/VCO_2_ slope VAT, minute ventilation/carbon dioxide production slope calculated from the start to the anaerobic threshold; bpm, breaths per minute; L, liters; VE, minute ventilation; m, meters; reps, repetitions; cm, centimeters; kg, kilograms.

When comparing baseline testing in CR patients with normal (*n* = 13) and abnormal (*n* = 16) resting spirometry, there was a significant difference in the percent of predicted peak VO_2_ (74.0[68.5–76.5]% v 62.0 [53.0–71.8]%; *p* = 0.02) and peak dominant hand grip (13.6 [9.5–17.9] kg vs. 9.1 [7.3–11.8] kg; *p* = 0.01). There was no significant difference in VE/VCO_2_ slope, 6-minute walk distance, or sit-to-stand repetitions. When comparing the change from the initial to the post-testing, patients with abnormal baseline spirometry had no significant difference in the change from initial to post-CR CPET variables, sit to stand, arm curl repetitions, or sit and reach.

When performing a subanalysis of those who completed in-person CR (*n* = 21) and those who completed a component of virtual CR (*n* = 8) there was no significant difference between the groups in the change from initial to post CR spirometry.

**Figure 3:**
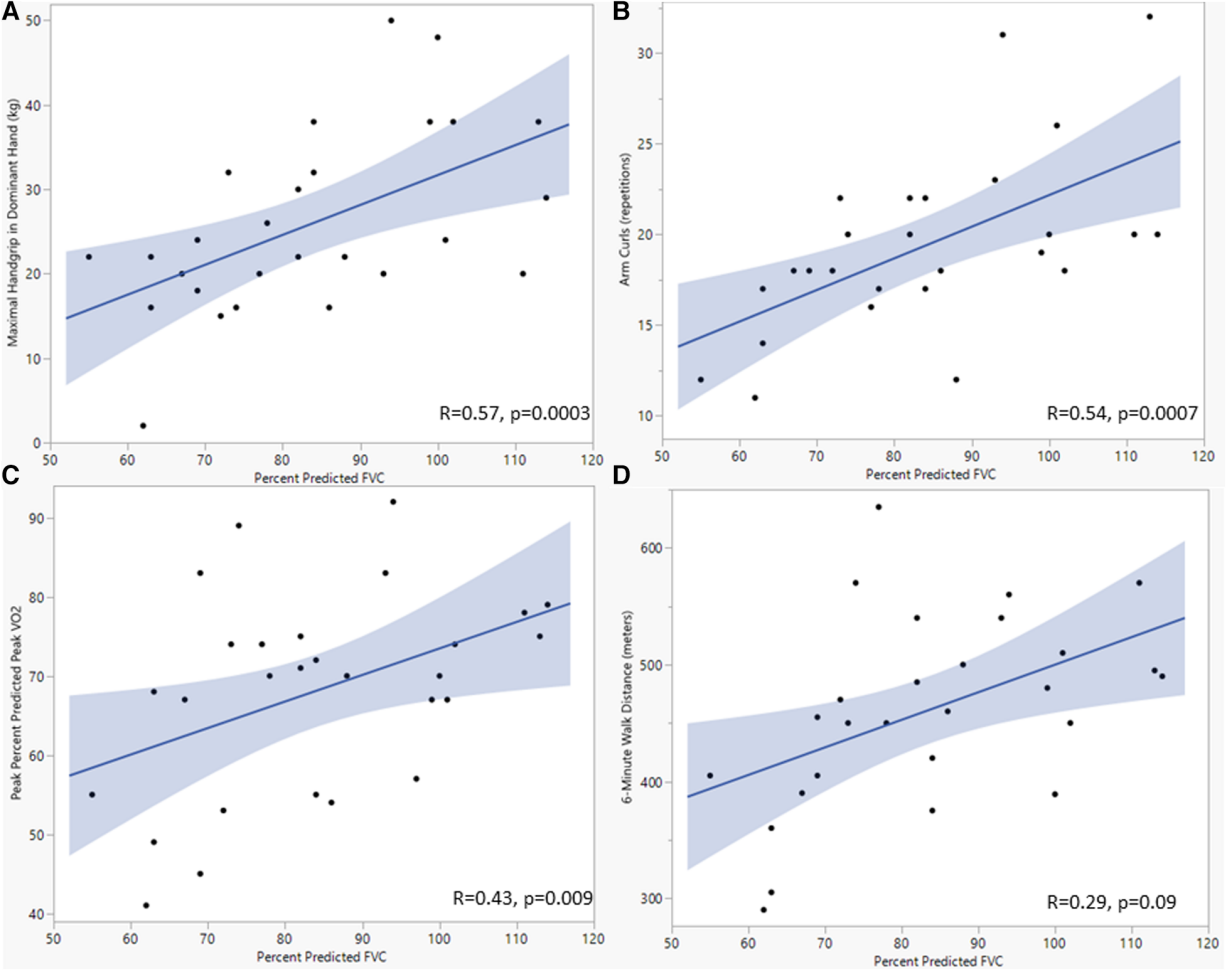
Scatterplots showing the relationship between forced vital capacity and maximal dominant handgrip (**A**), arm curl repetitions (**B**), percent predicated peak oxygen consumption (**C**), and 6-minute walk distance (**D**). Analyses used univariate linear regression, with FVC as the dependent variable. *P*-value <0.05 was considered significant. FVC, Forced vital capacity; kg, kilogram; Vo_2_, oxygen consumption.

When assessing for associations between spirometry and functional CR measures, FVC was associated with percent predicted peak VO_2_ (*r* = 0.43, *p* = 0.009) and peak tidal volume (*r* = 0.43, *p* = 0.01) (Table 4). Percent predicted FVC was associated with several functional CR outcomes, including increased peak VO_2_ (*r* = 0.43, *p* = 0.009), increased hand grip strength (*r* = 0.57, *p* = 0.0003), and increased arm curl reps (*r* = 0.54, *p* = 0.007) (Figure 3). Percent predicted FEV_1_ was associated with peak tidal volume (*r* = 0.49, *p* = 0.002) percent predicted peak VO_2_ (*r* = 0.33, *p* = 0.03), increased hand grip strength (*r* = 0.48, *p* = 0.02), and increased arm curl repetitions (*r* = 0.36, *p* = 0.02). Higher peak VE was associated with higher hand grip strength (*r* = 0.71, *p* < 0.0001), but resting VE was not associated with hand grip strength (*r* = 0.026, *p* *= *0.1). The total number of prior sternotomies was negatively correlated with percent predicted FVC (*r* = −0.43, *p* = 0.04), percent predicted FEV_1_ (*r* = −0.47, *p* = 0.02), percent predicted peak VO_2_ (*r* = −0.39, *p* = 0.04), and peak respiratory rate (*r* = −0.43, *p* = 0.03).

**Table 4:** Associations between forced vital capacity and forced expiratory volume at one second with cardiopulmonary exercise test and functional cardiac rehabilitation outcomes.

	FVC (%)		FEV_1_ (%)	
	R	*P* value	R	*P* value
Peak minute ventilation	0.45	0.005	0.43	0.007
Peak respiratory rate	−0.16	0.4	−0.3	0.1
Peak tidal volume	0.43	0.01	0.49	0.002
Peak VO_2_ (%)	0.43	0.009	0.33	0.03
6-minute walk	0.29	0.09	0.1	0.5
Dominant handgrip	0.57	0.0003	0.54	0.0007
Arm curl	0.54	0.0007	0.36	0.02
Sit to stand	0.25	0.1	0.18	0.3
Sit & reach	0.11	0.6	−0.03	0.9

Analyses performed with Spearman for non-parametric data. *P*-value <0.05 was considered significant. FVC, Forced vital capacity; FEV_1_, forced expiratory volume at one second; Vo_2_, oxygen consumption.

## Discussion

This study measured the baseline pulmonary function and dynamic components of ventilation in children and young adults enrolled in a clinical CR program. Patients with a history of CHD and prior surgery had worse baseline spirometry compared to those without heart disease, but resting spirometry and measures of dynamic ventilatory function did not significantly change following CR for any groups. Despite the lack of change in resting pulmonary function or dynamic ventilatory response to exercise, the cohort managed to improve both strength and aerobic fitness. Resting FVC and FEV_1_ were both associated with various CR outcomes including hand grip and 6-minute walk distance. Lastly, the number of prior sternotomies was associated with resting spirometry data.

This data implies that pulmonary improvements are not the primary driver for improved fitness in youth enrolled in clinical CR and rather these improvements are largely driven by improvements in either cardiac, vascular, or musculoskeletal function. Improvements in cardiovascular function following exercise training have been documented previously for young patients with heart disease (5, 12). Unfortunately, as clinical CR has not been widely utilized in pediatric hospitals, the mechanisms for these improvements in fitness and skeletal muscle strength are incompletely understood. Among these gaps in knowledge, little is known about the role of the pulmonary system in improving fitness and functional outcomes in young populations with heart disease undergoing CR.

Children and adults with CHD often have abnormal baseline restrictive lung disease likely secondary to a combination of previous median sternotomy and as a consequence of their underlying cardiac physiology on lung development. As studies in healthy children demonstrate resting spirometry and exercise ventilatory measures may be improved with exercise therapy, identifying if CR can improve lung function in this patient population is an important and understudied question. While assessment of pulmonary function following exercise-based intervention research in patients with CHD is minimal, Hedlund EL et al. previously demonstrated in a cohort of 25 patients with a Fontan circulation that resting pulmonary improvements are possible, which they hypothesized as secondary to improvements in lung volume and flow rates after aerobic exercise training (20). Our findings differ as both baseline spirometry and dynamic pulmonary and ventilatory measures (peak VE, tidal volume, respiratory rate, and VE/VCO_2_ slope) all remained unchanged. A potential explanation for these differences is that we reported resting spirometry as a percent of predicted as opposed to in absolute values, which are expected to increase in a largely pubertal population secondary to somatic growth. Additionally, the exercise stimulus was different in both studies, possibly leading to different pulmonary adaptations. Exercise training protocols with more of an overt training focus on respiratory muscles and breathing may result in different adaptations and should be explored. On the other hand, both cohorts were small, and these findings should be confirmed in a larger sample.

While measures of resting pulmonary function and dynamic components of ventilation did not improve with CR in our cohort, there were meaningful associations with resting spirometry and functional CR. Notably, a higher percent predicted FVC was associated with dominant peak handgrip, arm curl repetitions, and percent predicted peak VO_2_. This association implies that those young patients with heart disease and more normal lung volumes were able to perform many of the main functional outcomes measured by CR. This is supported by Smith et al. (21), who demonstrated an association between larger lung volumes and handgrip strength in patients with CHD. Another possible mechanism for these associations is that significant chest wall musculoskeletal strength is required for normal spirometry and ventilation and that these measures of CR and spirometry are all indirect measures of total skeletal muscle mass. To assess the effects of CR on resting pulmonary capacity more comprehensively, future studies could measure maximum voluntary ventilation, maximum inspiratory and expiratory pressure, and the diffusing capacity for carbon monoxide. These tests would determine the impact of CR on changes in respiratory muscle strength and alveolar gas exchange separately.

While the findings largely don't support significant improvements in resting pulmonary function and dynamic components of ventilation following CR, there are several important implications to these findings. For one, our findings support the hypothesis that resting pulmonary function may not be able to be improved upon using conventional CR techniques and timing. Studies of athletes and physically active adolescents and young adults demonstrate larger lung volumes on spirometry compared with healthy sedentary controls (22–25). The athletes with the highest lung volumes are swimmers where years of training positively correlated with lung volumes in some studies (26–28). On the other hand, studies examining spirometry in young athletes before and after training sessions limited to several months do not consistently demonstrate significant changes in volumes (29–31). Similarly, studies in adults who were previously sedentary, and who then began exercise show that while regular exercise may attenuate age-related decreases in resting lung volumes and exercise ventilatory capacity, lung volumes are not increased from pre-exercise values (32–35).

These findings support the concept that regular aerobic exercise during childhood and early adolescence may increase ventilatory capacity through adaptive growth only during a limited window until the individual reaches maturity. In our study where 78% of patients were ≥21 years old, these patients were likely already mature, or the exercise regimen was not long enough to impact lung growth. This should be confirmed in a younger cohort of patients with CHD prospectively focusing on respiratory muscle strengthening and aerobic conditioning. If this is correct, then extra care should be taken to preserve pulmonary function in this population with a high prevalence of lung disease. These measures should include aggressive smoking cessation counseling and community-based measures to reduce air pollution. Additionally, while the improvements in aerobic fitness as measured by peak VO_2_ do not appear to be attributed to resting pulmonary function and dynamic components of ventilation improvements, this means that training emphasis should be on the cardiac and musculoskeletal systems. Children should also be encouraged to engage in regular aerobic exercise at an early age able to stimulate lung growth. This is especially important in patients with previous cardiac surgery as they often have more pulmonary abnormalities on spirometry than those without surgery. Musculoskeletal training has been shown to increase skeletal muscle mass, including cardiac mass which can improve cardiac stroke volume (36). Other cardiac changes to optimize function can include addressing residual cardiac lesions, medication compliance, and regular surveillance in addition to fitness training. As CR did not result in improvements in pulmonary function, additional study should be performed for other treatment modalities in the care for those with underlying lung disease.

There were several limitations to our study. The cohort of patients included for analysis presented with various pathologies, and therefore varying degrees of cardiopulmonary dysfunction. Our study was limited in sub-analysis due to its small sample size, therefore this variability introduced additional uncertainty when interpreting pulmonary outcomes. This is a limitation that results from few high-volume clinical CR centers in pediatric hospitals. It remains unclear if those with more severe pulmonary pathology demonstrated a greater change in pulmonary outcome measures, therefore future studies should consider controlling for pulmonary co-morbidities during analysis. The lack of changes seen in the small subset who completed CR may not apply to the patients who did not complete CR. Additionally, the retrospective nature of this study could potentially introduce unknown biases and limitations in data collection, which is an inherent limitation in these types of studies. Lastly, the studied focused on possible pulmonary changes over a relatively short time frame (≥12 weeks), additional study should be performed to see if a longer duration exercise stimulus results in pulmonary adaptations in this population.

In conclusion, CR leads to improvements in peak aerobic capacity, but not in spirometric measures of pulmonary function or in dynamic ventilatory changes during exercise. Pulmonary function does appear to correlate with overall functional outcomes, including peak VO_2_ and measures of strength. Prior median sternotomy is associated with worse baseline pulmonary function, which is not improved following CR. Further research is required to elucidate associations between pulmonary function and functional and psychosocial outcomes.

## Data Availability

The raw data supporting the conclusions of this article will be made available by the authors, without undue reservation.
